# Research on a Visually Assisted Efficient Blind-Guiding System and an Autonomous Shopping Guidance Robot Arm Adapted to the Complex Environment of Farmers’ Markets

**DOI:** 10.3390/s25123785

**Published:** 2025-06-17

**Authors:** Mei Liu, Yunhua Chen, Jinjun Rao, Wojciech Giernacki, Zhiming Wang, Jinbo Chen

**Affiliations:** 1 Shanghai Key Laboratory of Intelligent Manufacturing and Robotics, School of Mechatronic Engineering and Automation, Shanghai University, Shanghai 200444, China; mliu@shu.edu.cn (M.L.); yunhuachen@shu.edu.cn (Y.C.); jjrao@shu.edu.cn (J.R.); suwangzm@shu.edu.cn (Z.W.); 2 Belt and Road Joint Laboratory on Measurement and Control Technology, Huazhong University of Science and Technology, Wuhan 430074, China; 3 Institute of Robotics and Machine Intelligence, Faculty of Control, Robotics and Electrical Engineering, Poznan University of Technology, Piotrowo 3a, 60-965 Poznan, Poland; wojciech.giernacki@put.poznan.pl

**Keywords:** high-precision mapping, efficient navigation, robotic arm guidance, autonomous shopping, visual aid

## Abstract

It is great challenge for visually impaired (VI) people to shop in narrow and crowded farmers’ markets. However, there is no research related to guiding them in farmers’ markets worldwide. This paper proposes the Radio-Frequency–Visual Tag Positioning and Automatic Detection (RFTPAD) algorithm to quickly build a high-precision navigation map. It combines the advantages of visual beacons and radio-frequency signal beacons to accurately calculate the guide robot’s coordinates to correct its positioning error and simultaneously perform the task of mapping and detecting information. Furthermore, this paper proposes the A*-Fixed-Route Navigation (A*-FRN) algorithm, which controls the robot to navigate along fixed routes and prevents it from making frequent detours in crowded aisles. Finally, this study equips the guide robot with a flexible robotic arm and proposes the Intelligent-Robotic-Arm-Guided Shopping (IRAGS) algorithm to guide VI people to quickly select fresh products or guide merchants to pack and weigh products. Multiple experiments conducted in a 1600 m^2^ market demonstrate that compared with the classic mapping method, the accuracy of RFTPAD is improved by 23.9%. What is more, compared with the general navigation method, the driving trajectory length of A*-FRN is 23.3% less. Furthermore, the efficiency of guiding VI people to select products by a robotic arm is 100% higher than that through a finger to search and touch.

## 1. Introduction

According to WHO statistics, there are currently about 253 million people with visual impairment worldwide, including 35.2 million blind people [1]. Although markets are often places where the visually impaired (VI) shop for essentials, most urban farmers’ markets, especially Chinese wet markets, are characterized by crowds of pedestrians, large spaces, and narrow aisles. It is worth noting that in such a complex environment with no tactile paving, it is a considerable challenge for VI people to walk and shop. Currently, most of the existing indoor guidance studies focus on family rooms, shopping malls, and subway stations [2,3], while there is no research related to farmers’ markets.

Researchers generally employ Simultaneous Localization and Mapping (SLAM) to control indoor guide robots to build navigation maps. In large-scale spaces, it is necessary to combine SLAM with fixed tag technology to supplement environmental information to improve mapping accuracy, such as Bluetooth beacons, Wi-Fi signalers, and Radio-Frequency Identification (RFID) tags [4,5,6]. Ahmetovic et al. [7] developed a shopping mall guide system based on Bluetooth beacon positioning. They employed a multivariate regression method to analyze the impact of shopping mall environments and positioning errors on the visually impaired walking along a planned route and proposed a series of technologies to improve the accuracy of the probabilistic positioning algorithm. Ivanov et al. [8] pasted RFID tags on the doors of hospital wards in order to enable the guide system equipped with tag readers to quickly read the room location information stored in them and correct their own posture. Gomes et al. [9] installed a series of visual tags, Bluetooth beacons, and Near-Field Communication (NFC) tags at different locations in a supermarket and designed a large supermarket guide system with a Wi-Fi positioning method to assist VI individuals in independently searching for products in a supermarket. However, the above common tags are not suitable for application in farmers’ markets. For example, the positioning accuracy of RFID and NFC tags is not high; the routing of Bluetooth and Wi-Fi beacons is cumbersome; and visual tags are easily damaged by water stains and blocked by pedestrians.

The path planning algorithms in existing guide methods are basically direct copies of autonomous driving methods. However, unlike normal people who randomly search for products, VI people prefer to shop along fixed routes to a few familiar stalls in farmers’ markets. Common fixed-route navigation methods include electromagnetic tracking, magnetic tape tracking, infrared tracking, visual tracking, etc. [10]. Škrabánek et al. [11] designed a magnetic-signal-based positioning system that applies low-cost magnetic tapes as cruising routes, detects the strength of a magnetic signal through a three-axis magnetometer sensor, and controls the robot’s forward movement and turning. Cruz et al. [12] designed a restaurant service robot based on infrared tracking, utilizing a black line as the robot’s cruising route in the restaurant, and controlling the robot’s forward movement and turning through a line-tracking algorithm. Nevertheless, the above methods are not suitable for farmers’ markets with complex environments—the cost of laying out and maintaining electromagnetic tracking tracks is high, infrared tracking tracks are easily contaminated by pedestrians’ soles, and these tracking methods do not have the function of avoiding pedestrians.

An autonomous shopping robot is a highly intelligent guide device consisting of a navigation car and a robotic arm. It can independently search and pick up target products for VI people and bring products back to them. Pauly et al. [13] designed a mobile robot for supermarkets and shopping malls. After a customer places orders for a desired product through a shopping interface, it immediately navigates to the target shelf, then identifies the target product by radio-frequency tags, and finally controls a robotic arm to pick up the product and put it into the shopping basket. Nechyporenko et al. [14] designed a product-picking robot arm for online shopping. They integrated the centroid normal method into a dual-arm robot system with two grippers, providing a practical solution to the robot picking problem in unstructured environments. Experimental tests showed that this method can control the robot arm to accurately pick up various products of different shapes. Although there are some research results on autonomous shopping robots, most of them are aimed at supermarkets rather than farmers’ markets. Additionally, these robots usually need to be equipped with a high-precision, high-load, and high-cost robotic arm, which is difficult for lower-paid VI people to afford.

According to the above analysis, it can be concluded that the mapping and navigation methods for guide robots in farmers’ markets are different from general guide methods. Fully considering the characteristics of the geometric layout and product features of farmers’ markets, this paper proposes a Radio-Frequency–Visual Tag Positioning and Automatic Detection (RFTPAD) algorithm to quickly build a high-precision navigation map. It improves mapping accuracy by combining RFID tag positioning and visual positioning methods to automatically calibrate the robot’s posture and improves efficiency by employing a classification model to automatically detect and record a large amount of product information. Furthermore, this paper proposes an A*-Fixed-Route Navigation (A*-FRN) algorithm to prevent guide robots from frequently taking long detours in crowded aisles. It plans a unique fixed navigation route for each large stall and controls the guide robot to walk along this route every time it navigates. Finally, this paper equips a guide robot with a robotic arm and proposes an Intelligent-Robotic-Arm-Guided Shopping (IRAGS) algorithm that combines deep learning, kinematic solving, and rapidly exploring random tree methods to intelligently control the robotic arm to guide VI people to quickly select fresh products.

## 2. Experimental Equipment

**Figure 1:**
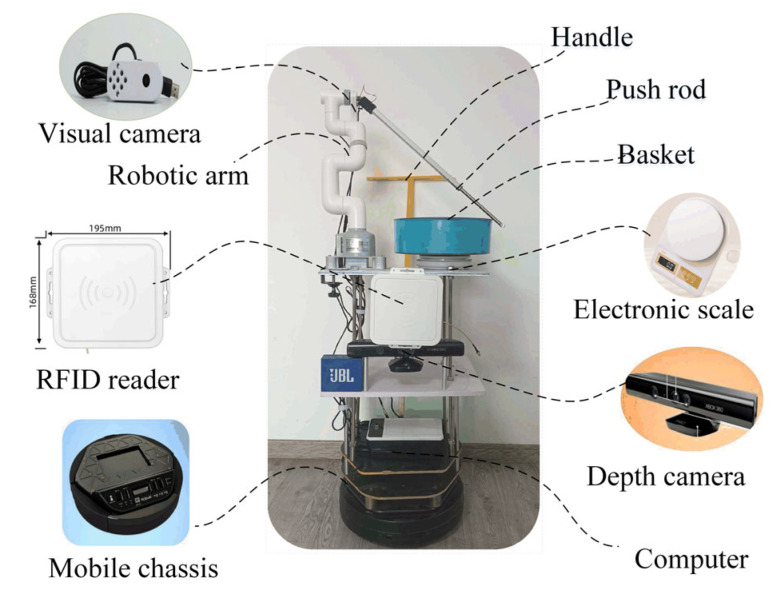
Hardware composition of guide robot.

Figure 1 shows the hardware devices of the market guide robot designed in this paper, which consists of two parts—a navigation car and robotic arm guidance system. Its parameters are as follows: length × width × height is 35.4 × 35.4 × 160 cm^3^, the weight is 10.5 kg, and the speed is 0.65 m/s.

The navigation car is mainly composed of the following components: mobile chassis, depth camera, industrial computer, RFID reader, and wooden handle. Among them, the mobile chassis can move flexibly in narrow shopping aisles and measure a robot’s posture with encoders and gyroscopes; the depth camera can scan the depth information of geometric environments for functions such as building a map and obstacle avoidance; the RFID reader is used to read the information in the RFID tags; and the wooden handle is used to guide VI people to move forward and turn.

The guidance system is mainly composed of the following components: a robotic arm, visual camera, electric push rod, varistor, and electronic scale. Among them, the robotic arm has the ability of guiding VI people or merchants to select product; the retractable electric push rod can point out products; the visual camera is used to locate and identify products, pedestrians, merchants, and VI peoples; and the varistor is used to detect whether the VI people’s finger is holding the robotic arm.

The model number, manufacturer, city, and country of above equipment are shown in Table 1.

The cost of the guide robot is about 21,600 RMB, where the robotic arm is 6000 RMB, mobile chassis is 7000 RMB, depth camera is 1000 RMB, industrial computer is 5000 RMB, RFID reader and tags are 1200 RMB, electric push rod is 1000 RMB, and other parts are 400 RMB.

## 3. High-Precision Mapping and Intelligent Navigation Strategy

### 3.1. Synchronous High-Precision Mapping and Automatic Product Detection

As mentioned in the Introduction, employing fixed tag technology to supplement environmental information is a common method to improve positioning accuracy. RFID positioning has the characteristics of low cost, easy deployment, and strong anti-pollution ability. Assume that the received signal strength index of the RFID tag read by the tag reader is RSSI at time t. The RSSI value can be converted to the distance value according to Equation (1) [15,16]:

(1) $$d=10^{\left(\mathrm{ABS}\left(\mathrm{RSSI}\right)-A\right)/\left(10\times n\right)}$$

where *A* is the signal strength value when the tag is 1 m away from the reader, *A* = −45.8; n is the signal attenuation coefficient, *n* = 3; and ABS is the symbol for the absolute value.

Although RFID positioning technology is relatively mature, the RFID tag is easily blocked and reflected by obstacles in the environment and interfered with by wireless signals. The measured RSSI value is inconsistent with the actual RSSI, which leads to non-negligible errors in the RFID positioning process.

The principle of the visual positioning method based on artificial beacons is to place a beacon with known information at a fixed point and use a visual camera to detect the relative position of the robot and the beacon. Visual cameras have the characteristics of rich information collection, good robustness, and strong anti-interference. Nevertheless, the visual beacons placed in farmers’ markets are easily covered by stains or blocked by pedestrians [17].

This paper combines the advantages of artificial beacons and the RFID localization method to design a RFTPAD algorithm, which can accurately calculate the robot’s position and correct accumulated positioning errors in robot mapping processes. The algorithm process is as follows: RFTPAD firstly employs a general Cartographer method [18,19] to control the guide robot to build a map. Simultaneously, it runs the RFID reader to receive the nearest RFID tag signal and obtain its posture stored in advance. Subsequently, it controls the robot automatically approaching the RFID tag. Finally, it applies the visual positioning method to calculate the robot’s posture and correct the accumulated positioning error.

Additionally, due to the wide variety of commodities in farmers’ markets, it is time-consuming and labor-intensive to manually count their names and coordinates. Thus, this paper sets up RFTPAD to call a classification model to automatically detect a product’s categories and coordinates while it controls the guide robot to build map.

#### 3.1.1. Position Calibration Method Based on RFID Tags

**Figure 2:**
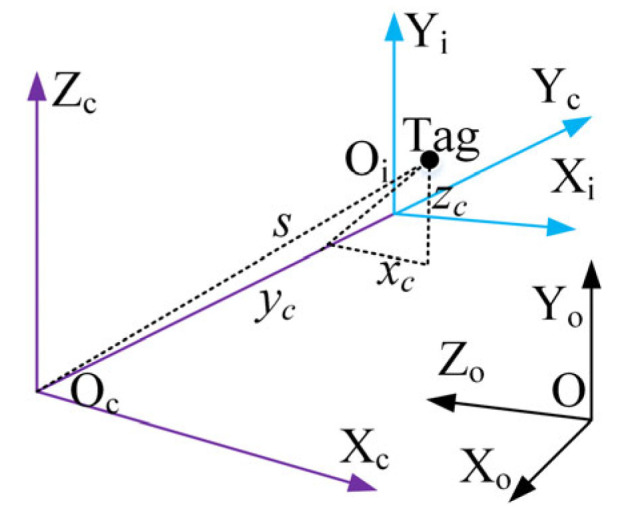
Three coordinate systems based on the principles of perspective geometry.

This paper sets three coordinate systems based on the principle of perspective geometry [20], as shown in Figure 2, setting a two-dimensional image physical coordinate system O_i_X_i_Y_i_, whose origin O_i_ is the image plane center, and the X_i_-axis and Y_i_-axis are parallel to the two vertical edges of the image, respectively; setting a three-dimensional camera coordinate system O_c_X_c_Y_c_Z_c_, whose origin O_c_ is the camera center, Y_c_-axis coincides with the camera optical axis and intersects with the image plane center O_i_, and X_c_-axis and Z_c_-axis are parallel to the two image vertical edges, respectively; and setting map coordinate system OX_o_Y_o_Z_o_, whose origin O is the starting point of the map, X_o_-axis and Y_o_-axis are parallel to the ground plane, and Z_o_-axis is perpendicular to the ground plane.

Given the distance *s* between the camera center and RFID tag, as well as the image coordinates (*x_i_*, *y_i_*) of the RFID tag center, it can be inferred that the coordinates (*x_c_*, *y_c_*, *z_c_*) of the RFID tags relative to the robot in the camera coordinate system are as follows:

(2) $$\begin{array}{c} x_{c}=x_{i}\\ z_{c}=y_{i}\\ y_{c}=\sqrt{s^{2}-{x_{i}}^{2}-{y_{i}}^{2}} \end{array}$$

Assuming the transformation matrix from the camera coordinate system to the map coordinate system is as follows:

(3) $$T=\left[\begin{array}{cccc} \cos\theta & -\sin\theta & 0 & x\\ \sin\theta & \cos\theta & 0 & y\\ 0 & 0 & 1 & 0\\ 0 & 0 & 0 & 1 \end{array}\right]$$

where *x* and *y* are the robot coordinates on the map, and *θ* is the robot angle relative to origin O.

Then, the coordinates *N_o_* (*x_o_*, *y_o_*, *z_o_*) of the RFID tag in the map coordinate system can be calculating as follows:

(4) $$\begin{array}{lll} \left[\begin{array}{l} x_{o}\\ y_{o}\\ z_{o}\\ 1 \end{array}\right] & = & \left[\begin{array}{cccc} \cos\theta & -\sin\theta & 0 & x\\ \sin\theta & \cos\theta & 0 & y\\ 0 & 0 & 1 & 0\\ 0 & 0 & 0 & 1 \end{array}\right]\left[\begin{array}{l} x_{c}\\ y_{c}\\ z_{c}\\ 1 \end{array}\right]\\ & = & \left[\begin{array}{c} x_{i}\cos\theta -\sqrt{s^{2}-{x_{i}}^{2}-{y_{i}}^{2}}\sin\theta +x\\ x_{i}\sin\theta +\sqrt{s^{2}-{x_{i}}^{2}-{y_{i}}^{2}}\cos\theta +y\\ y_{i}\\ 1 \end{array}\right] \end{array}$$

Given that the real coordinates of the RFID tags in the map coordinate system are *N* (*x_n_*, *y_n_*, *z_n_*), if there is no positioning error during the robot mapping process, then *N_o_* and *N* are the same. Thus, the following equation holds true:

(5) $$\left[\begin{array}{l} x_{o}\\ y_{o}\\ z_{o}\\ 1 \end{array}\right]-\left[\begin{array}{l} x_{n}\\ y_{n}\\ z_{n}\\ 1 \end{array}\right]=\left[\begin{array}{c} x_{i}\cos\theta -\sqrt{s^{2}-{x_{i}}^{2}-{y_{i}}^{2}}\sin\theta +x-x_{n}\\ x_{i}\sin\theta +\sqrt{s^{2}-{x_{i}}^{2}-{y_{i}}^{2}}\cos\theta +y-y_{n}\\ y_{i}-z_{n}\\ 0 \end{array}\right]=\left[\begin{array}{l} 0\\ 0\\ 0\\ 0 \end{array}\right]$$

Due to *θ* = arctan (*y*/*x*), a binary equation about the unknown quantities *x* and *y* can be obtained from (5):

(6) $$\left\{\begin{array}{l} x_{i}\cos\theta -\sqrt{s^{2}-{x_{i}}^{2}-{y_{i}}^{2}}\sin\theta +x-x_{n}=0\\ x_{i}\sin\theta +\sqrt{s^{2}-{x_{i}}^{2}-{y_{i}}^{2}}\cos\theta +y-y_{n}=0 \end{array}\right.$$

By solving this equation, the exact robot coordinates (*x*, *y*) in the map coordinate system can be determined.

#### 3.1.2. Detection of Product Information Based on Transfer Learning

Transfer learning based on deep learning can quickly obtain a new model with high identification accuracy by simply adjusting the parameters of a pre-trained model [21]. This study employs transfer learning to build a product identification model. The pre-trained model selected in this paper is MobileNetV2, which is a convolutional network model with the ability of accurately identifying 1000+ types of objects [22]. The network architecture of the model trained by transfer learning is consistent with the pre-trained model. Table 2 shows the network structure configuration diagram of the product identification model.

**Figure 3:**
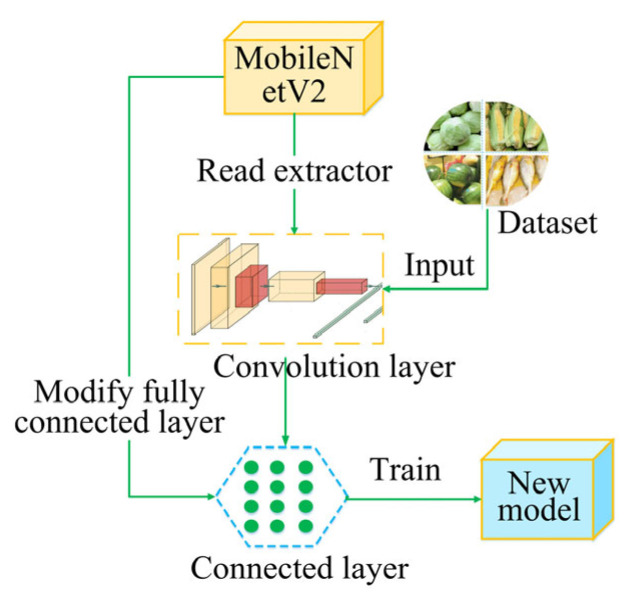
The process of building the product model.

The process of building the product identification model is shown in Figure 3. Firstly, RFTPAD calls the network of MobileNetV2 as the feature extractor for the new model; then, it modifies the fully connected layer of MobileNetV2 into a 394-category classifier; and finally, it inputs the self-made dataset and trains only the classifier parameters to obtain a preliminary new model. The dataset contains various samples of common products in farmers’ markets, and each sample contains 5000 pictures.

### 3.2. Fixed-Route Navigation Strategy Based on A*-FRN Method

As mentioned in the introduction, general navigation methods are not suitable for farmers’ markets with complex geometry and shopping environments, as well as the shopping habits of VI people. By referring to the A* method [23,24] and classical fixed-route navigation methods [10,25], this paper proposes an A*-FRN algorithm to prevent robots from frequently taking detours in crowded aisles. Compared with general fixed-route cruising methods, it does not require laying a tracking route and has the advantages of low cost, high accuracy, and flexibly avoiding pedestrians.

#### 3.2.1. A*-FRN Algorithm

**Figure 4:**
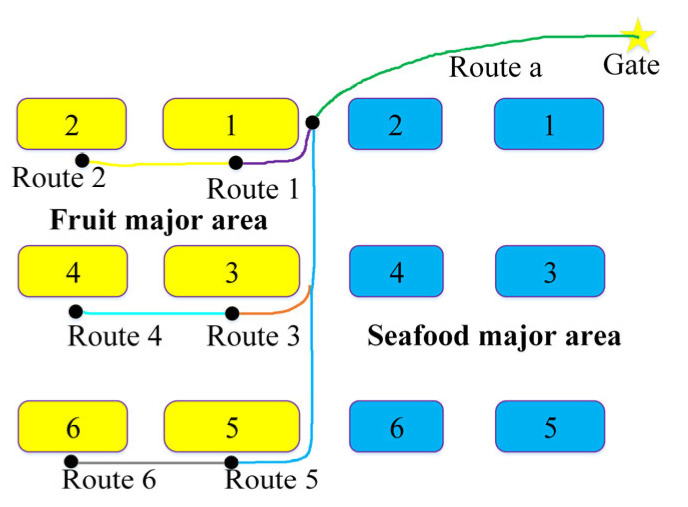
The fixed route planned by A*-FRN from the gate to the fruit major area. The star represents the market gate. Each rectangle represents a stall, and the same color rectangles represent that they belong to the same areas. The numbers represent the serial numbers of the stalls in each product area. The lines represent the routes planned by A*-FRN, and the dot represents the end point of the route.

When there are too many pedestrians in aisles causing guide robots to fail to avoid obstacles, the A* method will abandon the optimal global navigation route and plan a longer route. To address this problem, this study proposes the A*-FRN algorithm, which plans a unique fixed navigation route for each large stall and controls the guide robot to walk along this route every time it navigates. The process of A*-FRN is as follows: A*-FRN firstly divides a map into major areas such as vegetable, fruit, meat, seafood, grain, and oil ones according to product categories and names each large stall in each major area in numerical order (see the numbers in the rectangles in Figure 4); subsequently, it employs the A* method to plan the shortest route from the market gate to the No. 1 large stall in each major area and names it in alphabetical order (see green line in Figure 4); furthermore, it plans the shortest route from the No. 1 large stall in each major area to other large stalls in this area and names each route in the large stall number N (see the non-green line in Figure 4); and finally, by combining these routes, all the fixed routes can be obtained from the gate to all the large stalls in each major area.

**Figure 5:**
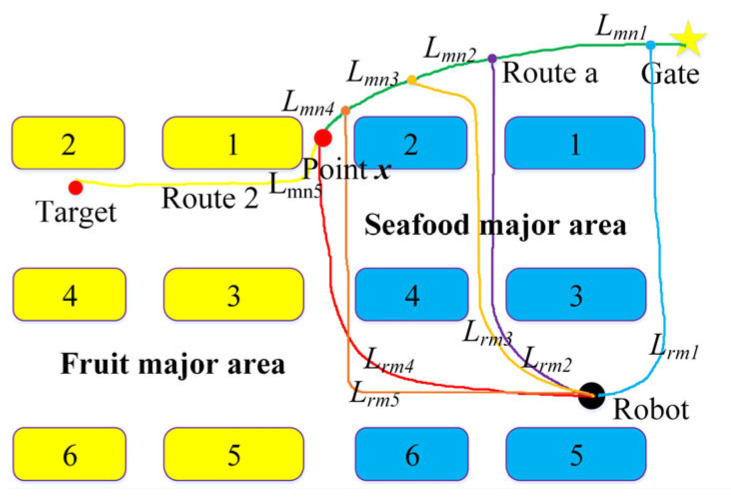
The schematic diagram of the A*-FRN controlling robot. The lines represent the routes planned by A*-FRN. The black dot represents robot and other dots represent the end point of the routes.

Figure 5 is a schematic diagram of A*-FRN controlling the robot navigating to target stall 2 in fruit major areas. It first calculates the optimal point *x* from the robot’s location to fixed route a; subsequently, it employs the PID method [26,27] to control it to navigate to point *x*; then, it controls it to drive along route a and route 2 to target stall 2; and finally, it utilizes the A* method to navigate to the position where the target product is located. The method for calculating point *x* is as follows:

1. Calculate the route length *L_rm_* from the robot to any point *m* on fixed route a with the A* method;
2. Then, calculate the route length *L_mn_* from point m along route a and route 2 to target stall 2;
3. Add *L_rm_* and *L_mn_* to obtain the total length *L_smn_* = *L*_rm_ + *L*_mn_;
4. Compare *L_smn_* with the smallest value. The corresponding point *m* is optimal point *x*.

Particularly, since pedestrians may stand on the planned fixed route, it is impossible for the guide robot to walk completely according to the planned route. This study sets up A*-FRN to employ the DWA method [28] to control the robot to avoid obstacles. Then, the robot does not need to return to the fixed route as long as it is in the same channel as the fixed route. Subsequently, A*-FRN employs the A* method to plan a dynamic route for it to move forward, which starts from the robot’s current position and ends at the same point as the fixed route in the current aisle. Additionally, the A*-FRN algorithm will control the guide robot to stop moving forward if the channel is completely blocked. It continues to control it to navigate along a fixed route until the passage is clear and there is enough space for the robot to pass through.

#### 3.2.2. A* Method

A* is a classical path planning method, which has the advantages of a simple search path and a rapid response to the environment. A* divides a map into N nodes and calculates the cost value from the current node to the next node through a cost function [29,30]. The node with the smallest cost value is the next search node, and ultimately connecting all the selected nodes will result in an optimal path. The cost function of the A* algorithm is as follows:

(7) $$f\left(n\right)=g\left(n\right)+h\left(n\right)$$

where *f*(*n*) represents the cost value from the initial node to the target node, *g*(*n*) represents the actual cost value from the initial node to the current node, and *h*(*n*) represents the estimated cost value from the current node to the target node.

### 3.3. Intelligent Guidance and Autonomous Shopping Strategy Based on Robotic Arm

Unlike general guide robots that only have simple navigation and obstacle avoidance functions, this study equips a 6-degree-of-freedom flexible robotic arm with light weight (1.1 kg) and low cost (6000 RMB) for a guide robot in a farmers’ market and designs an IRAGS algorithm for controlling it. It not only could intelligently guide VI people to quickly select fresh fruits and vegetables but also could combine with a navigation car to enable autonomous shopping.

#### 3.3.1. Intelligent Guidance for VI People to Select Fresh Products

The process of the IRAGS algorithm guiding VI people to select fresh fruits and vegetables is as follows: firstly, it employs a visual camera to identify relatively fresh target products; subsequently, it controls the robotic arm to touch VI people’s fingers and prompts them through voice to grasp the end effector of the robotic arm; furthermore, it controls the robotic arm to pull VI people’s fingers to the surface center of the target product; and finally, after VI people grab the product, it will control the robot arm to pull their finger to place the selected product on the robot’s built-in electronic scale.

(a) Control the motion of the robotic arm

**Figure 6:**
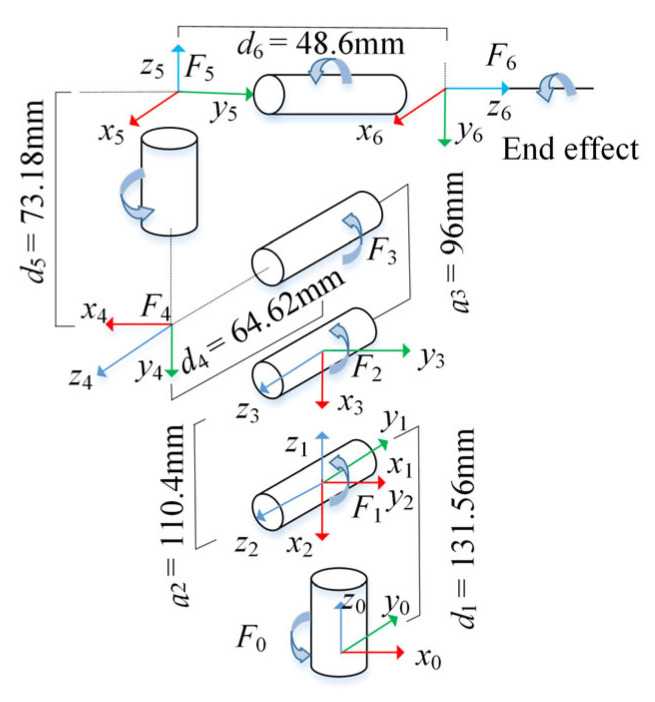
D-H coordinate system of mycobot_280_M5 robot.

The Denavit–Hartenberg (D–H) method is a classic method for kinematic modeling and analysis of robotic arms, which can describe the coordinate system relationship and geometric coefficients between adjacent links [31]. This paper applies the D–H method to construct the link coordinate system of the mycobot_280_M5 robotic arm, as shown in Figure 6.

According to the link coordinate system constructed above, the D–H parameters of each joint of mycobot_280_M5 can be calculated, as shown in Table 3. *a_i_* is the link length, which represents the distance from the *z*_i_ axis to the *z_i_*_+ 1_ axis along the *x*_i_ axis. *α_i_* is the link twist, which represents the angle of rotation from the *z_i_* axis to the *z_i_*_+ 1_ axis along the *x_i_* axis. *d_i_* is the link offset, which represents the distance from the *x_i_*_− 1_ axis to the *x_i_* axis along the *z_i_* axis. *θ_i_* is the joint angle, which represents the angle of rotation from the *x_i_*_− 1_ axis to the *x_i_* axis along the *z_i_* axis.

The D–H coordinate transformation matrix between the two adjacent links is as follows:

(8) Ti  i−1=RotZi−1,θiTransZi−1,diTransXi,aiRotXi,αi=cosθi−sinθicosαisinθisinαiaicosθisinθicosθicosαi−cosθisinαiaisinθi0sinαicosαidi0001

According to the parameters in Table 3 and Equation (8), the transformation matrix between the two adjacent links can be calculated as follows:

(9)   T10=c10−s10s10c10010d10001     T21=c2−s200s2c20000100001   T32=c30−s3a3c3s30c3a3s30−1000001T43=c4−s400s4c400000d40001    T54=c50−s50s50c500−1000001   T65=c6−s600s6c600001d60001

where *c_i_* is the abbreviation of cos*θ_i_*, and *s_i_* is the abbreviation of sin*θ_i_*.

The D–H transformation matrix of the end of the robot arm relative to the base coordinate system is as follows:

(10) T60= 10T21T32T43T54T65T=nxoxaxpxnyoyaypynzozazpz0001

where (*n_x_*, *n_y_*, *n_z_*) ^T^, (*o_x_*, *o_y_*, *o_z_*) ^T^, and (*a_x_*, *a_y_*, *a_z_*) ^T^ represent the posture vectors of the end of the robot, and *p_x_*, *p_y_*, and *p_z_* represent the position vectors of the end of the robot. Their specific values are as follows:

$$n_{x}=c_{6}\left[-c_{1}s_{5}s_{23}+c_{5}\left(s_{1}s_{4}+c_{1}c_{4}c_{23}\right)\right]+s_{6}\left(c_{4}s_{1}-c_{1}c_{23}s_{4}\right)$$

$$n_{y}=c_{6}\left[-s_{1}s_{5}s_{23}+c_{5}\left(c_{1}s_{4}+s_{1}c_{4}c_{23}\right)\right]+s_{6}\left(c_{4}c_{1}+s_{1}c_{23}s_{4}\right)$$

$$n_{z}=s_{4}s_{6}s_{23}-c_{6}\left(c_{23}s_{5}+c_{4}c_{5}s_{23}\right)$$

$$o_{x}=-s_{6}\left[-c_{1}s_{5}s_{23}+c_{5}\left(s_{1}s_{4}+c_{1}c_{4}c_{23}\right)\right]+c_{6}\left(c_{4}s_{1}-c_{1}c_{23}s_{4}\right)$$

$$o_{y}=-s_{6}\left[-s_{1}s_{5}s_{23}+c_{5}\left(c_{1}s_{4}+s_{1}c_{4}c_{23}\right)\right]+c_{6}\left(c_{1}c_{4}-c_{23}s_{1}s_{4}\right)$$

$$o_{z}=s_{6}\left(c_{23}s_{5}+c_{4}c_{5}s_{23}\right)+c_{6}s_{4}s_{23}\;\;\;a_{x}=-s_{5}\left(s_{1}s_{4}+c_{1}c_{4}c_{23}\right)+c_{1}c_{5}s_{23}$$

$$a_{y}=-s_{5}\left(c_{1}s_{4}+c_{4}c_{23}s_{1}\right)-c_{5}s_{1}s_{23}\;\;\;a_{z}=c_{4}s_{5}s_{23}-c_{5}c_{23}$$

$$p_{x}=c_{1}\left(a_{2}c_{2}+a_{3}c_{23}\right)-d_{6}\left[s_{5}\left(s_{1}s_{4}+c_{1}c_{4}c_{23}\right)+c_{1}s_{23}c_{5}\right]-c_{1}s_{23}d_{4}$$

$$p_{y}=s_{1}\left(a_{2}c_{2}+a_{3}c_{23}\right)-d_{6}\left[s_{5}\left(c_{1}s_{4}+s_{1}c_{4}c_{23}\right)+s_{1}s_{23}c_{5}\right]-s_{1}s_{23}d_{4}$$

$$p_{z}=d_{1}-c_{23}d_{4}-a_{2}s_{2}-a_{3}s_{23}-d_{6}\left(c_{5}c_{23}-c_{4}s_{5}s_{23}\right)$$

where *s*_23_ and *c*_23_ are the abbreviations of sin(*θ*_2_ + *θ*_3_) and cos(*θ*_2_ + *θ*_3_), respectively.

The inverse kinematic solution is to calculate the value of each joint variable based on the known position of the end of the robot arm. The central axes of the last three joints of the mycobot_280_M5 robotic arm intersect at one point, which satisfies the Pieper criterion. Therefore, the inverse kinematics of mycobot_280_M5 can be solved by an analytical method. The specific calculation method can be found in reference [32,33].

**Figure 7:**
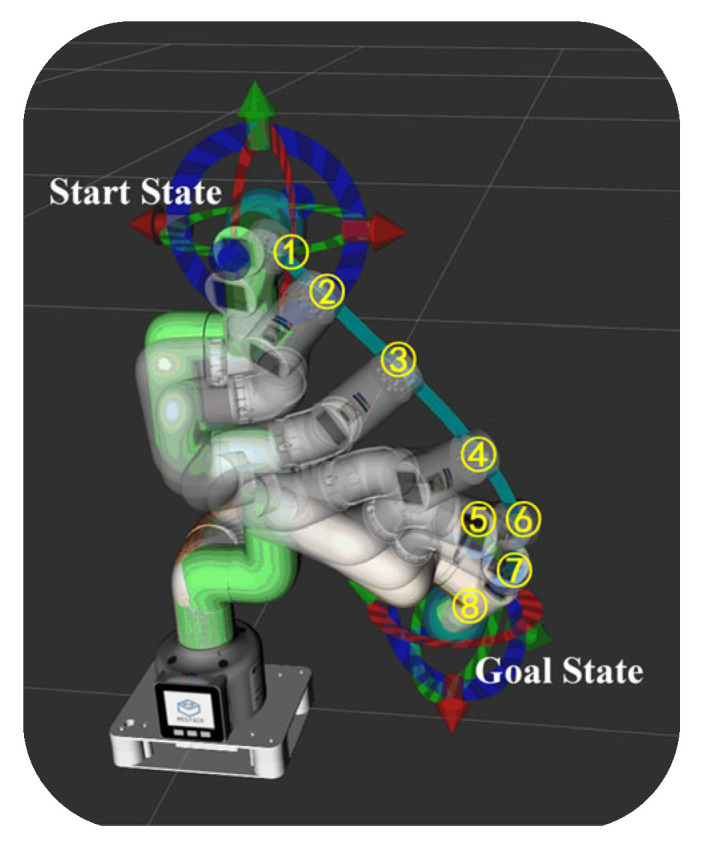
The process of the IRAGS planning route and controlling the robot arm in ROS. The serial numbers ➀–➇ represent the 8 moments when the robot moves along the planned route.

The rapidly exploring random tree is a sample collection-based route planning method with advantages of high efficiency, simplicity, and great completeness, and it is suitable for various types of multi-degree-of-freedom robotic arms [34]. This paper sets up IRAGS to apply the rapidly exploring random tree to accurately control the motion of the robotic arm. Figure 7 shows IRAGS planning a route from the start point to the target point and controlling the robot arm moving along the route in the ROS (version is Kinetic Kame) simulation tool Rviz (version 1.12.17).

(b) Locating products and VI people’s fingers based on monocular visuals

**Figure 8:**
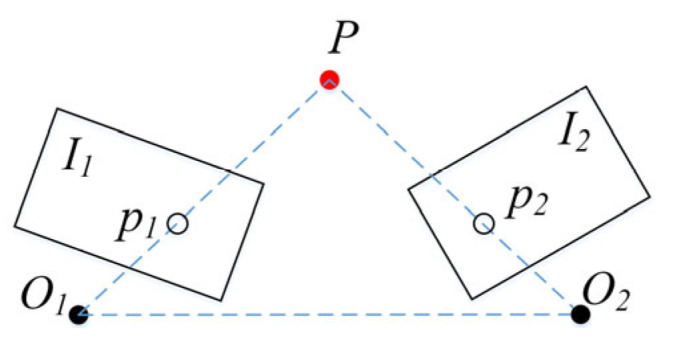
The schematic diagram of the triangulation method.

Monocular visual localization with advantages of low cost, low power consumption, and small computational complexity is commonly applied for locating object coordinates. This study sets the IRAGS algorithm to employ monocular visual localization based on the triangulation method [35,36] to detect the coordinates of VI people’s fingers and products. As shown in Figure 8, it determines the depth pixel point *P* by observing the angle between the same point at two different positions (*O*_1_, *O*_2_). After determining one side length and two angles of the triangle, the position of target point *P* can be calculated. Assuming the coordinate of *P* in the image physical coordinate system (see Section 3.1.1) is (*x*, *y*), its value can be directly obtained from the camera output. Assuming that the Y_c_ coordinate of *P* in the camera coordinate system (see Section 3.1.1) is *y*_c_, its value can be calculated by the triangulation method. Then, point *P* can be reached by controlling the robotic arm to simultaneously move the distance |*y_c_*| along the Y_c_-axis in the camera coordinate system and move the distances |*x*| and |*y*| along the X_i_-axis and Y_i_-axis in the image physical coordinate system, respectively.

(c) Identify fresh products based on transfer learning

This study employs transfer learning to build an identification model to distinguish fresh fruits and vegetables. The specific method is shown above in Section 3.1.2. Since the product classification model that has been trained in Section 3.1.2 can identify various types of fruits and vegetables, this study applies it as a pre-training model. The self-made training dataset contains 394 types of fresh fruit and vegetable samples with a smooth appearance, obvious color, and standard outline, as well as 394 types of fruit and vegetable samples in various un-fresh degrees (such as rotten, bumped, with obvious spots, etc.). This was obtained by web crawling, and there are 5000 pictures of each type of fruit and vegetable.

#### 3.3.2. Robot Autonomous Shopping Strategy

During peak shopping hours, the narrow aisles may be so crowded that it is difficult for VI people to pass through. The IRAGS algorithm can also control robots to shop autonomously, saving the physical strength of VI people and meeting their needs for remote shopping.

**Figure 9:**
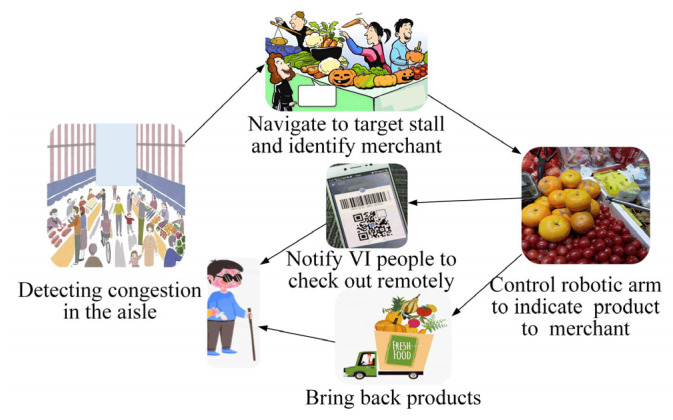
The process of controlling the robot to shop autonomously with IRAGS.

The schematic diagram of controlling robots to shop autonomously with the IRAGS algorithm is shown in Figure 9. Firstly, it runs the pedestrian detection model based on Faster R-CNN [37] to detect the number of pedestrians within 3 m in front of the robot. IRAGS will run the A*-FRN method to control a robot to navigate autonomously if the number exceeds the threshold T. Subsequently, when the robot arrives at a destination, IRAGS immediately employs the above monocular visual localization method to detect the coordinates of each person near the target stall. If someone is standing behind the target stall and his coordinates are the most similar one to the target stall coordinates, A*-FRN determines that he is the target merchant. Furthermore, it employs the PID [26,27] method to control the robot to approach the target merchant and controls the end effector of the robotic arm to indicate the target product for him and reminds him by voice to place the product on the robot’s built-in electronic scale. Then, IRAGS sends the calculated total price and the merchant’s payment code to VI people, notifying him to check out. Finally, it drives the robot to bring products back to the VI people.

## 4. Experiment and Discussion

**Figure 10:**
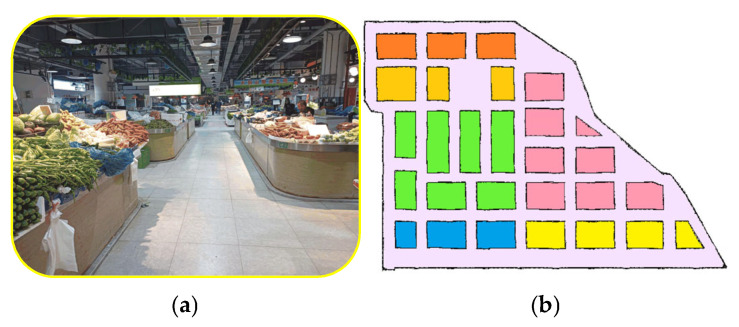
Trial site and navigation map: (**a**) Qixiang Farmers’ Market, address: No. 3399, Qilianshan Road, Baoshan District, Shanghai, China; (**b**) navigation map. The colored polygons correspond to stalls and other pink areas correspond to aisles. The same color polygons represent that they belong to the same areas.

Figure 10a shows the farmers’ market selected for the experiment, with its detailed address description. The area of the market is about 1600 m^2^, and the aisle width is 2 m. Figure 10b is a navigation map constructed by the RFTPAD algorithm, where the colored polygons correspond to stalls and other pink areas correspond to shopping aisles. Particularly, the two stalls facing each other in the same channel are 2 m apart, and the signal range of the RFID tag selected in this study is 8 m. Therefore, it can meet the requirements of the RFTPAD algorithm by installing an RFID tag only on one of the two stalls. A total of 56 tags were installed in this farmers’ market.

### 4.1. Comparative Analysis of Mapping Accuracy and Efficiency with RFTPAD

#### 4.1.1. Accuracy Analysis Between RFTPAD and Cartographer Algorithm

In this trial, eight coordinate points P_m_ (*x*_m_, *y*_m_) were randomly selected and input into the robot’s navigation target one by one. The maps constructed by RFTPAD and classical Cartographer algorithms [18,19] were used for navigation, respectively, and the navigation data were recorded to compare their accuracy. Assuming that the absolute navigation error *σ*_1_ is the absolute value of the difference between the robot’s theoretical navigation distance *S_m_* and the actual driving distance *S_n_*, then that is *σ*_1_ = |*S_m_* − *S*_n_|, and the average error value *σ*_2_ is the arithmetic mean of eight absolute errors.

The navigation error data obtained from experimental statistics are shown in Table 4. The third and fourth columns represent the actual driving distance of the robot with the Cartographer algorithm and RFTPAD, respectively, while the fifth and sixth columns represent the absolute navigation error of the robot with the Cartographer algorithm and RFTPAD, respectively. Comparing the fifth and sixth columns, it can be observed that as the navigation distance increases, the error of the map constructed with RFTPAD is consistently smaller than that of those constructed with the Cartographer algorithm. Furthermore, by calculating the data in the table, the average error values of the robot with RFTPAD and the Cartographer algorithm are *σ*_2*R*_ = 0.051 m and *σ*_2*C*_ = 0.067 m, respectively. (*σ*_2*C*_ − *σ*_2*R*_)/*σ*_2*C*_ × 100% = 23.9%, which means that compared to the Cartographer algorithm, RFTPAD reduces the map error by 23.9%.

#### 4.1.2. Synchronize Detection of Product Information

**Figure 11:**
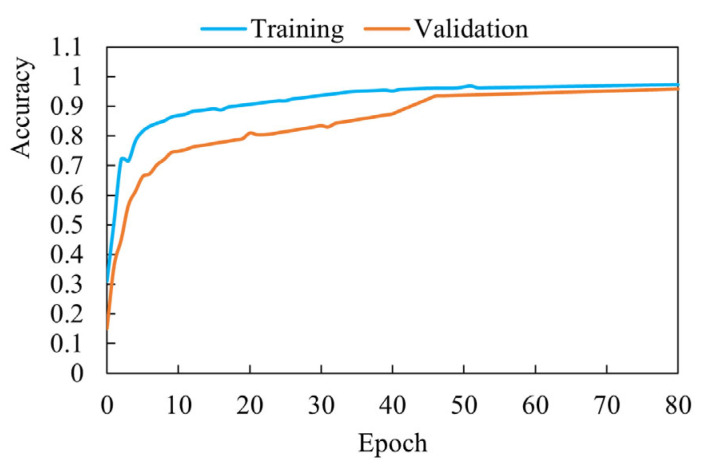
Training and validation accuracy of the product identification model. The horizontal axis represents the training epoch, and the vertical axis represents the recognition accuracy.

The experiment first inputs the image dataset into the product identification model for training. The dataset contains 394 category samples of common products in farmers’ markets, and each sample contains 5000 pictures. Figure 11 shows the accuracy change during model training. Observing the right side of the figure, it can be found that the recognition accuracy of the new model finally stabilized at 95% when the model was trained to convergence.

**Figure 12:**
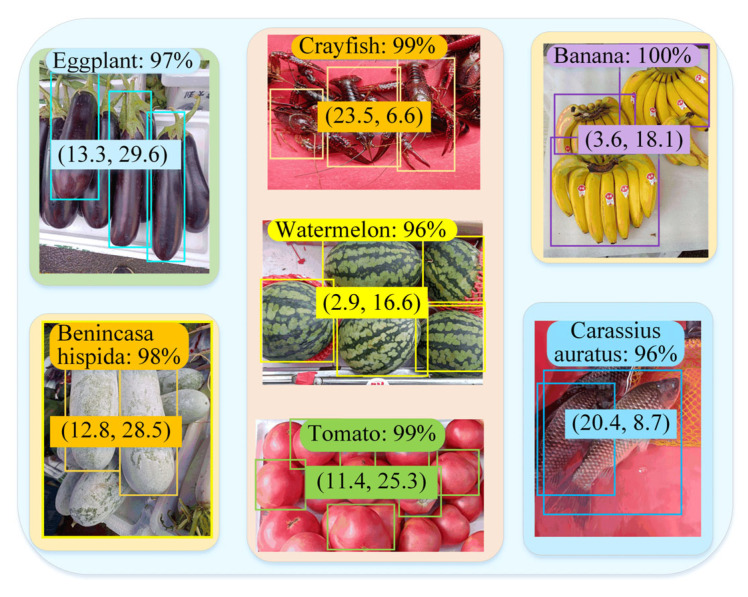
Products detected by RFTPAD.

Figure 12 shows some products detected by RFTPAD while the robot is building a map. The percentage represents the identification accuracy, and the numbers in brackets represent the product coordinates (unit is m). By comparing the product’s actual categories in the figure with the results identified by RFTPAD, it can be seen that the identified results are completely correct, and its accuracy rate reaches more than 95%.

Subsequently, the experiment set up the robot to record product information (category and coordinates) with RFTPAD’s automatic detection method and manual counting method, respectively. A total of six trials were conducted, and the area of the trial site was gradually increased. The steps of the manual counting method are as follows: the mapping personnel manually control the guide robot to go to each type of product, manually check its coordinates, and record them as the product coordinates.

**Figure 13:**
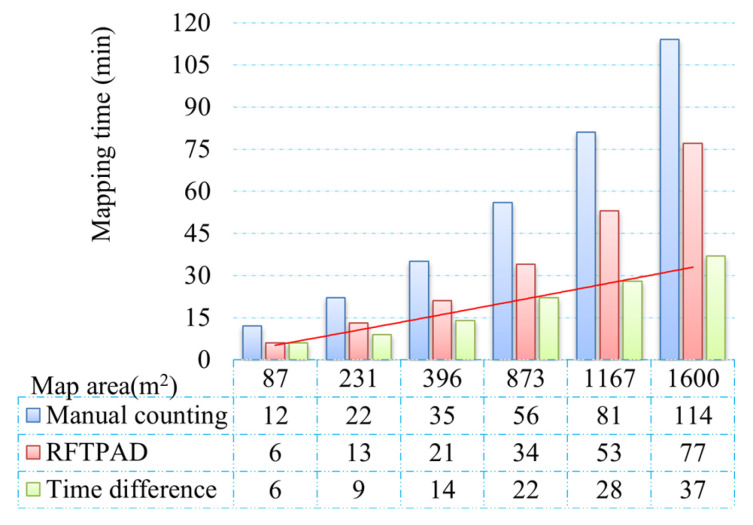
The time taken to record product information with RFTPAD and the manual counting method, respectively.

Figure 13 is a statistical chart showing the time taken to detect and record all the product information in the farmers’ markets with the above two methods, respectively. The horizontal axis represents the trial site area; the red and blue columns represent RFTPAD and the manual counting method, respectively; and the cyan column and red line represent their difference. By comparing the red and blue columns as a whole, it is obvious that no matter how large the trial site area is, the time spent with the RFTPAD algorithm is less than that with the manual counting method. By calculating the data in the figure, it can be concluded that the average time of RFTPAD is 36.3% less than that of the manual counting method. By observing the red trend line in the figure, it can be found that the difference in the time taken by the two methods gradually increases as the area of the trial site increases, which indicates that the RFTPAD algorithm has an obvious advantage in large farmers’ markets with a wide variety of products.

### 4.2. Fixed-Route Navigation Trial with A*-FRN Algorithm

**Figure 14:**
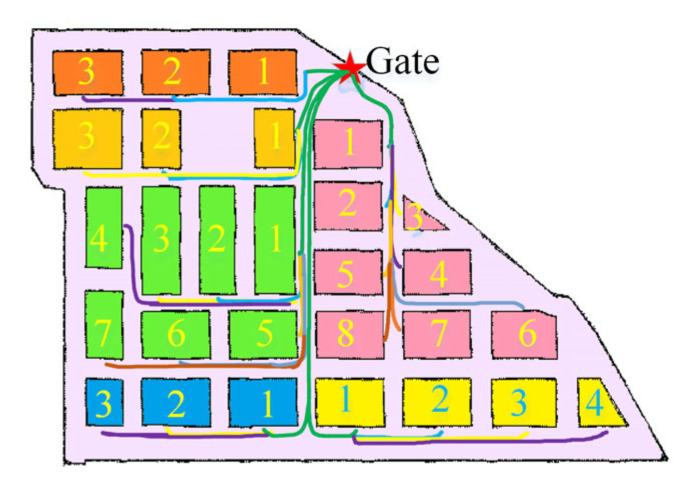
The numbers represent the serial numbers of the stalls in each product area. The fixed routes planned by A*-FRN. The red five-pointed star in the upper right corner represents the starting point of the route, and the colored lines represent the planned fixed route.

The colored lines in Figure 14 are the fixed routes planned by A*-FRN on a map. Among them, the green lines represent the fixed routes from the gate to the first large stall in each major area, totaling six routes; the other colored lines represent the fixed routes from the first large stall in each major area to the other large stalls in this area, totaling 22 routes; and there are a total of 28 routes.

#### 4.2.1. Analysis of Robot’s Detour Behavior When Aisle Is Crowded

**Figure 15:**
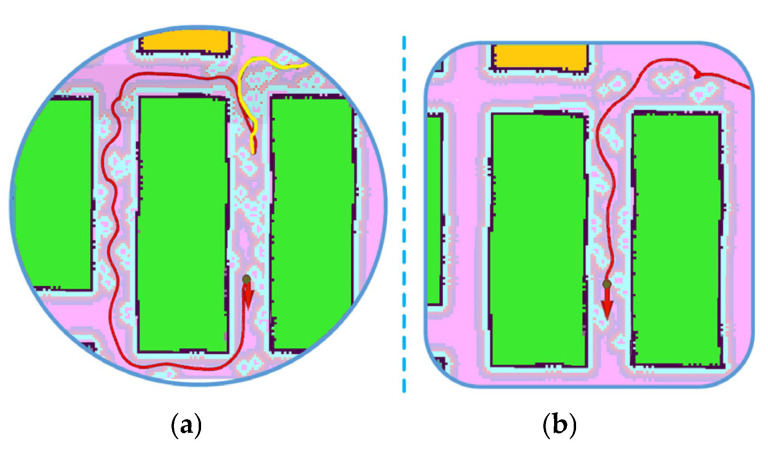
The detour behavior of the robot: (**a**) the robot’s trajectory under A*-DWA control; (**b**) the robot’s trajectory under A*-FRN control. The black dot represents the robot, the red arrow represents the destination, the blue circles represent the pedestrians detected by the robot, and the colored lines represent the robot’s trajectory.

The trial set up the guide robot to navigate in crowded aisles with the A*-FRN algorithm and the A*-DWA method [23,28,30] (A* and DWA algorithm, a classic navigation method combination), respectively. Figure 15 shows the robot’s driving trajectory with the above different methods. It can be seen from Figure 15a that the robot’s initial trajectory (see yellow line) was about to reach the destination, but then it turned away from the destination and took a long detour (see red line). The detour behavior of the robot with the A*-DWA method shows that it did not follow the optimal route. Compared with Figure 15a, it can be found that the trajectory line of the robot with A*-FRN in Figure 15b approaches the destination very directly, and the overall trajectory length is much shorter. By measuring the trajectory lines, it can be concluded that the driving trajectory of the robot with A*-FRN is about 8 m shorter than that of the robot with A*-DWA.

#### 4.2.2. Analysis of Robot’s Driving Trajectory Length When Aisle Is Crowded

Then, the trial set up the robot to navigate back and forth between the starting point and destination and employed the A*-FRN algorithm and A*-DWA method to control the robot to navigate, respectively, and it recorded its driving trajectory data. Each algorithm was tested 20 times, and the navigation distance was set to increase gradually. Furthermore, the experiment set the robot to run the A*-DWA method when it navigated from the starting point to the destination and run A*-FRN when it returned from the destination to the starting point.

**Figure 16:**
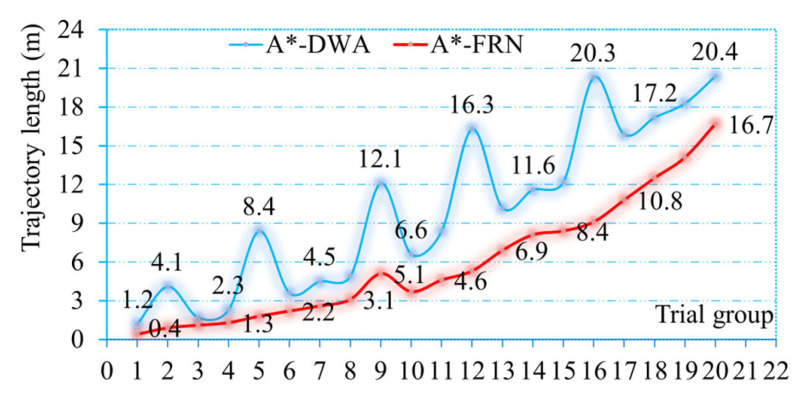
Robot’s driving trajectory length with different algorithm control, respectively.

The statistical results of the robot’s driving trajectory length are shown in Figure 16. The blue curve and red curve represent the robot’s driving trajectory length with the A*-DWA method and A*-FRN algorithm, respectively. It can be clearly observed that the blue curve is always above the red curve, which means that when the navigation target is the same, the robot’s overall driving length is shorter when it runs A*-FRN than when it runs A*-DWA. Moreover, it can also be observed that in the 2nd, 5th, 9th, 12th, and 16th trials, the ordinate values corresponding to the blue curve are significantly higher than those of the red curve. Based on the analysis above, we can conclude that the reason for the obvious gap between the two is as follows: when the guide robot runs A*-FRN to navigate, it can intelligently judge the crowded conditions and adopt the strategy of navigating along a fixed global route without giving up the originally planned shortest path. By calculating the data in the figure, it can be concluded that the average driving trajectory length of the robot with the A*-FRN algorithm is 23.3% less than that with the A*-DWA method.

### 4.3. Intelligent Guided and Autonomous Shopping Based on Robotic Arm

#### 4.3.1. Intelligent Selection of Fresh Products with the Assistance of a Robotic Arm

Calibration is a prerequisite for the precise control of the robotic arm. The experiment firstly calibrated the robotic arm and camera. This trial utilized the calibration program of mycobot_280_M5 to set the joint zero position and initialize the potential value of the motor. Then, the trial employed Zhang’s calibration method [38,39] to calibrate the camera mounted on the robotic arm. The camera intrinsic parameters and distortion parameters were calculated as follows:

$$\mathrm{Intrinsic}\;\mathrm{parameters}\;=\left[\begin{array}{ccc} 1193.35 & 0 & 634.72\\ 0 & 132.16 & 531.59\\ 0 & 0 & 1 \end{array}\right]$$

$$\mathrm{Distortion}\;\mathrm{parameters}\;=\left[\begin{array}{ccccc} 0.026 & 0.623 & 0.018 & -0.025 & -1.968 \end{array}\right]$$

**Figure 17:**
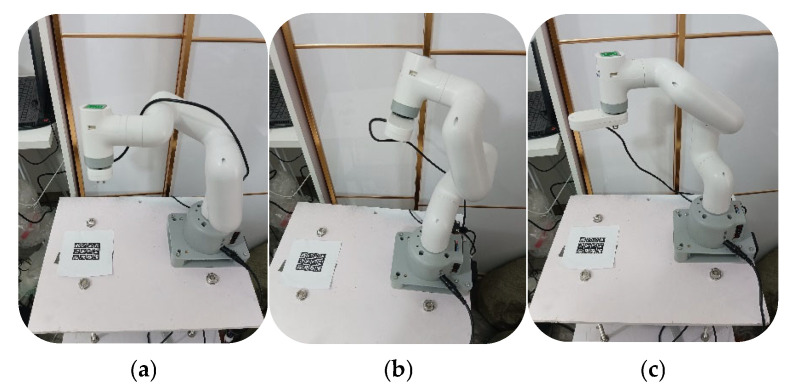
The process of the camera collecting calibration plate data at different positions: (**a**) camera is on the right of the calibration plate; (**b**) camera is on the lower right of the calibration plate; (**c**) camera is directly above the calibration plate.

Furthermore, the experiment performed hand–eye calibration on the robotic arm and camera and solved the transformation matrix between the camera coordinate system and the base coordinate system. The trial firstly utilized a camera mounted on a robotic arm to take multiple images of the same calibration plate in different postures. Subsequently, it calculated the geometric transformation of the robotic arm and camera between different image frames to obtain the transformation matrix. Figure 17 shows the process of the camera collecting calibration plate data at different positions.

The calculated hand–eye transformation matrix is as follows:

$$\left[\begin{array}{cccc} -0.853 & -0.033 & 0.006 & 0.042\\ 0.051 & -0.836 & 0.031 & 0.096\\ 0.006 & 0.063 & 0.981 & -0.043\\ 0 & 0 & 0 & 1 \end{array}\right]$$

**Figure 18:**
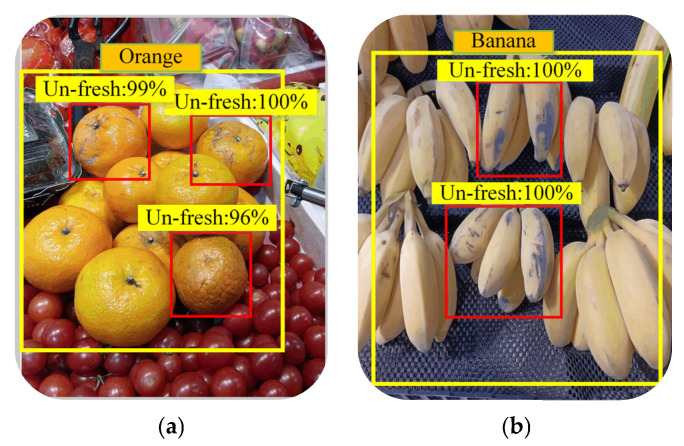

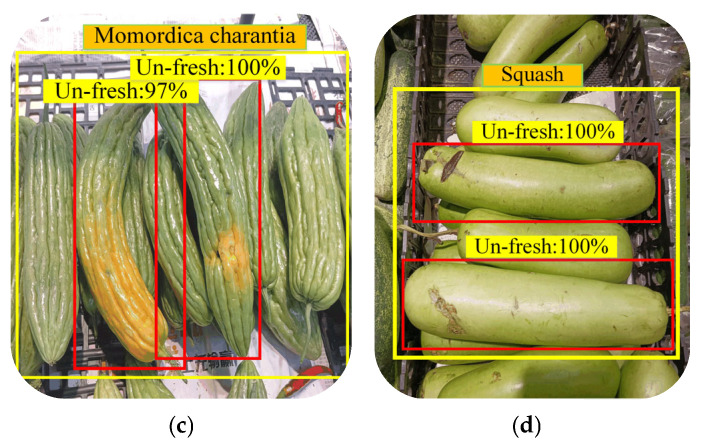
Un-fresher fruits detected by IRAGS: (**a**) detected oranges; (**b**) detected bananas; (**c**) detected momordica charantia; (**d**) detected squash. The red boxes represent the un-fresher fruits identified by the IRAGS algorithm, and the numbers above them represent recognition accuracy.

Subsequently, the experiment tested the IRAGS algorithm’s ability of accurately detecting un-fresh products. Figure 18 shows several target products identified by the IRAGS algorithm after the guide robot navigated to its destination. As can be seen from the figure, IRAGS correctly selected target products from various fruits with large yellow boxes and marked some un-fresher target products with small red boxes. After a careful observation of the products in small boxes, it was found that they have obvious black spots, an abnormal appearance color, and even damaged outer skin, while the target products not marked with small boxes basically have the characteristics of a smooth appearance, a bright color, and a standard outline. Particularly, the data in the figure show that IRAGS can identify non-fresh products with an accuracy of over 96%.

Next, the trial arranged the VI volunteers to select products with the general method (search and touch with fingers) and the assistance of a robotic arm, respectively, and it recorded the time it took to select the fresher products. Although the selected volunteers are completely blind, they have rich experience in selecting commodities. A total of eight trials were conducted, and 2 kg of a certain type of relatively fresh product was selected for each trial. The selected products were apples, bananas, small mangos, dates, potatoes, carrots, green peppers, and cherry tomatoes.

**Figure 19:**
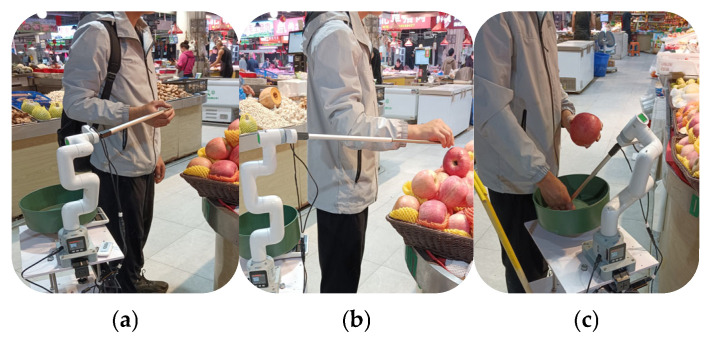
The process of the robot arm pulling VI people’s fingers to grab an apple: (**a**) the robotic arm located and touched the VI people’s fingers; (**b**) the robotic arm pulled the fingers to grab the product; (**c**) the robotic arm guided the fingers to place and weigh the product.

Figure 19 shows the process of the IRAGS controlled robotic arm to pull VI people’s fingers to pick an apple, where Figure 19a shows that IRAGS accurately located and controlled the retractable push rod to touch VI people’s fingers; Figure 19b shows that the robotic arm successfully pulled the VI people’s finger to touch the target product; and Figure 19c shows that the robotic arm guided the VI people’s fingers to place and weigh the selected product on the robot’s built-in electronic scale.

**Figure 20:**
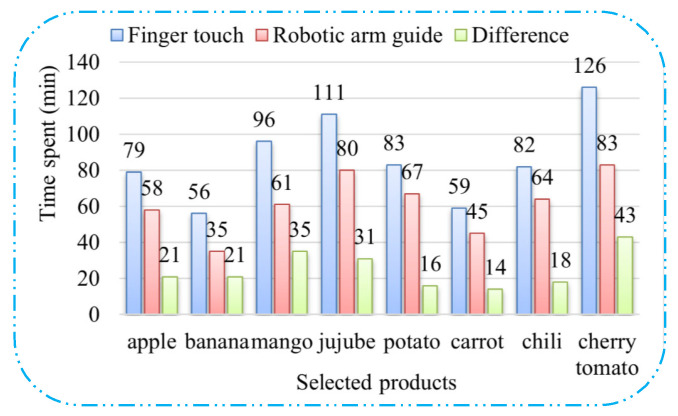
The time statistics of the VI people who selected products with the two methods.

Figure 20 shows the time statistics of the VI people who selected products with the general method and the assistance of the robotic arm, respectively. It can be clearly observed that the time spent by VI people in selecting products with the assistance of the robotic arm is always much less than that of the general method. Specifically, there is a significant difference in the time it took for VI people to select smaller items (mangos, cherry tomatoes, and jujubes) with the two different methods. Additionally, by calculating the data in the figure, it can be concluded that the average time spent by VI people to select various products with the assistance of the robotic arm is 50% of the time spent with the general method.

#### 4.3.2. Autonomous Shopping Based on Robotic Arm

The experiment first conducted multiple robot obstacle avoidance tests in aisles with different crowd densities to determine the threshold T. During the experiment, it was found that when the number of pedestrians within 3 m in front of the robot was greater than eight, the robot frequently turned to avoid pedestrians and even frequently changed the global navigation route. Therefore, the experiment set threshold T to eight.

**Figure 21:**
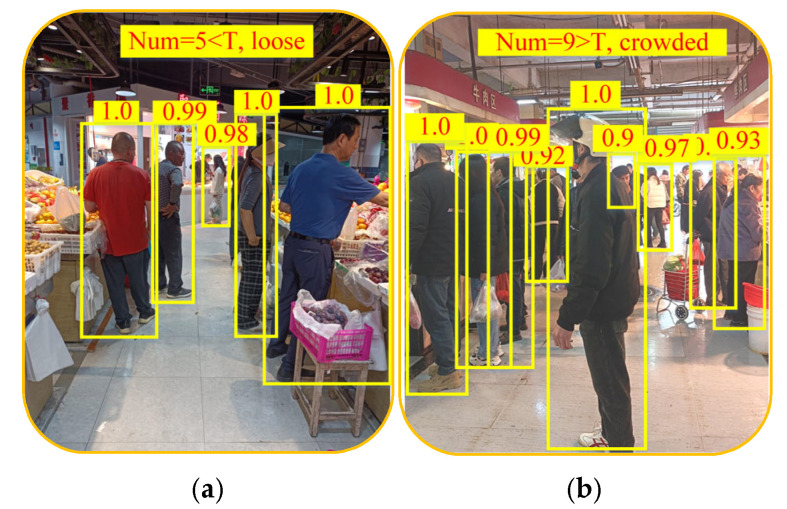
The congestion status in aisles detected by IRAGS. The yellow box represents the pedestrians identified by the IRAGS algorithm, and the number above it represents the recognition accuracy. Num at the top represents the number of identified pedestrians: (**a**) IRAGS Identifies pedestrians in loose aisle; (**b**) IRAGS identifies pedestrians in crowded aisle.

Subsequently, the trial arranged the guide robot to automatically detect the number of pedestrians within 3 m ahead. The two sub-images in Figure 21 show the congestion status in the aisles detected by the IRAGS algorithm. Among them, the number of pedestrians detected in Figure 21a is five, which is less than the threshold T, and IRAGS determined that this aisle is loose; on the contrary, the number of pedestrians detected in Figure 21b is nine, and IRAGS determined that this aisle is crowded. Furthermore, as can be observed from Figure 21, IRAGS correctly detected every pedestrian in the aisles and counted their number. This is consistent with the actuality, which proves that IRAGS can indeed accurately determine the congestion status in an aisle.

**Figure 22:**
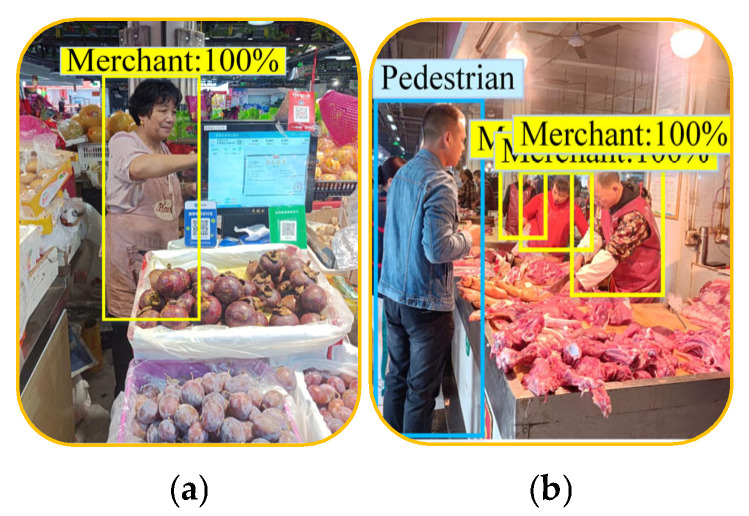
Merchants detected by IRAGS: (**a**) identified merchant without crowd interference; (**b**) identified merchants from the crowd.

When IRAGS determined that the aisle was crowded, it recommended that the VI people give an instruction to the robot to shop autonomously. Figure 22 shows the target merchants identified by IRAGS, with an identification accuracy of 100%. Furthermore, observing Figure 22b, it can be found that IRAGS accurately selected pedestrians and merchants, indicating that IRAGS can indeed accurately distinguish merchants from the crowd without being disturbed by pedestrians.

**Figure 23:**
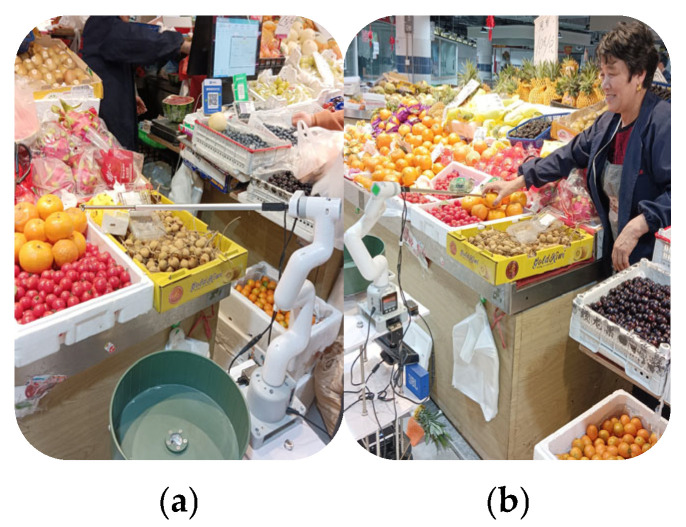
IRAGS guided the merchant to select and weigh the product: (**a**) the robotic arm pointed out the fresher target product; (**b**) the merchant grabbed the product according to the instruction of the robotic arm.

Then, the trial set up the guide robot with the control of IRAGS to autonomously approach the target merchant and remind them to select and weigh the product specified by the robotic arm. Figure 23a shows the IRAGS controlled robotic arm pointing out the relatively fresh target product for the merchant; Figure 23b shows the merchant grabbing the product according to the instruction of the robotic arm.

## 5. Conclusions

This paper systematically designed a novel guide robot for a farmers’ market, which has the capabilities of quickly building high-precision maps, fixed-path navigation, intelligent robotic arm guidance, and autonomous shopping.

Aimed at the complex dynamic environment of farmers’ markets, this study proposes the RFTPAD algorithm, which innovatively employs RFID-based artificial visual tags to calibrate a robot’s posture and applies deep learning to automatically detect and record a large amount of product information. Multiple trials conducted in a 1600 m^2^ market demonstrate that the accuracy of the map built with RFTPAD is 23.9% higher than that built with the classical Cartographer algorithm. What is more, the average time spent to detect products with RFTPAD is reduced by 36.3% compared with the manual counting method. Additionally, in terms of navigation, this study proposes the A*-FRN algorithm to control the robot to navigate along a fixed route, successfully preventing the robot from frequently taking long detours in a crowded farmers’ market. Compared with the general navigation method, the average driving trajectory length of the robot with A*-FRN is reduced by 23.3%.

Furthermore, in order to improve the robot’s intelligence, this paper innovatively equips the robot with a flexible robotic arm with the characteristics of light weight and low cost, and it specially designs the IRAGS algorithm to control it. In the robot shopping guide trials, the robotic arm successfully assisted VI people in selecting fresh products. Particularly, its efficiency was doubled compared with selecting products using fingers to search and touch. Additionally, in robot autonomous shopping trials, IRAGS successfully controlled the robotic arm to guide merchants to select and weigh products and automatically brought the remotely paid products back to VI people.

## Figures and Tables

**Table 1:** The model number, manufacturer, city, and country of the equipment on the guide robot.

Equipment Name	Model Number	Manufacturer	City and Country
Mobile chassis	Kobuki	Yujin Robot Co., Ltd.	Seoul, Republic of Korea
Depth camera	Kinect V1	Microsoft Corporation	Redmond, WA, USA
Computer	MN50	EVOC Intelligent Co., Ltd.	Shenzhen, China
RFID reader	NRD909	WYUA Co., Ltd.	Guangzhou, China
Robotic arm	mycobot_280_M5	Elephant Robotics Co., Ltd.	Shenzhen, China
Visual camera	cameraHolder_J6	Elephant Robotics Co., Ltd.	Shenzhen, China
Electric push rod	MNTL	LONGXIANG Co., Ltd.	Changzhou, China

**Table 2:** Network structure configuration of product identification model. t represents the dimensionality increase ratio of the 1 × 1 convolution in the Inverted Residuals structure, c represents the depth channel of the output feature matrix, n represents the number of bottleneck repetitions, and s represents the stride of the DW convolution in the first bottleneck.

Input	Operator	t	c	n	s
224^2^ × 3	conv2d	-	32	1	2
112^2^ × 32	bottleneck	1	16	1	1
112^2^ × 16	bottleneck	6	24	2	2
56^2^ × 24	bottleneck	6	32	3	2
28^2^ × 32	bottleneck	6	64	4	2
14^2^ × 64	bottleneck	6	96	3	1
14^2^ × 96	bottleneck	6	160	3	2
7^2^ × 160	bottleneck	6	320	1	1
7^2^ × 320	conv2d 1 × 1	-	1280	1	1
7^2^ × 1280	avgpool 7 × 7	-	-	1	-
1 × 1 × 1280	conv2d 1 × 1	-	k	-	

**Table 3:** The D–H parameters of mycobot_280_M5.

Joint *i*	Joint Angle *θ_i_* (°)	Link Offset *d_i_* (mm)	Link Length *a_i_* (mm)	Link Twist *α_i_* (°)
1	θ1	131.56	0	90
2	θ2	0	−110.4	0
3	θ3	0	−96	0
4	θ4	64.62	0	90
5	θ5	73.18	0	−90
6	θ6	48.6	0	0

**Table 4:** Comparison of navigation errors with RFTPAD and the Cartographer algorithm.

Group	*S_m_* (m)	*S_nC_* (m)	*S_nR_* (m)	*σ*_1*C*_ (m)	*σ*_1*R*_ (m)
1	15.121	15.152	15.097	0.031	0.024
2	22.353	22.396	22.389	0.043	0.036
3	29.468	29.419	29.429	0.049	0.039
4	34.644	34.583	34.597	0.061	0.047
5	38.517	38.595	38.456	0.078	0.061
6	41.376	41.460	41.439	0.084	0.063
7	46.243	46.154	46.178	0.089	0.065
8	55.689	55.587	55.723	0.102	0.074

## Data Availability

The data presented in this study are available on request from the author Yunhua Chen.
